# A rare presentation of male breast atypical papillary lesion

**DOI:** 10.1016/j.radcr.2026.03.070

**Published:** 2026-05-07

**Authors:** Kiarash Soltani, Rozhan Esmaeili Benvidi, Samane Rashidi, Mahdi Taghdiri, Elham Keshavarz

**Affiliations:** a Department of Radiology, Shahid Beheshti University of Medical Sciences, Tehran, Iran; b Department of Pathology, Imam Khomeini Hospital, Tehran University of Medical Sciences, Tehran, Iran; c Department of Radiology, Imam Khomeini Hospital, Tehran University of Medical Sciences, Tehran, Iran; d Department of Radiology, Mahdiyeh Hospital, Shahid Beheshti University of Medical Sciences, Tehran, Iran

**Keywords:** Male breast, Atypical papillary lesion, Hemorrhagic nipple discharge

## Abstract

Papillary breast lesions are a heterogeneous group of lesions with epithelial origin, composed predominantly of papillae, and they are particularly rare in men. Atypical papillary lesions are rare epithelial tumors, characterized by cytologic or architectural features that are not clearly benign but may fall short of definitive malignancy. Herein, we present a case of a 51-year-old man admitted with painless, hemorrhagic discharge from the left nipple. Ultrasonography revealed an intraductal solid mass in the left breast, along with mild bilateral gynecomastia. Core needle biopsy (CNB) subsequently confirmed an atypical papillary lesion. This case highlights the importance of imaging and tissue biopsy in establishing an accurate diagnosis, due to their wide spectrum of histological presentations from benign to malignant, which should be well differentiated.

## Introduction

Papillary breast lesions predominantly affect women, though they can rarely occur in men. In men and postmenopausal women, papillary lesions are more likely to be malignant [1]. Papillary neoplasms are mainly composed of papillae, each consisting of a fibrovascular core, lined by epithelium that may be accompanied by an MEC layer, based on tumor type. Depending on the epithelial features, papillary lesions are categorized as benign, atypical, and malignant. Moreover, the presence, distribution, and histopathology of MECs should be evaluated. This can be done through immunohistochemical (IHC) staining with a panel of MEC markers such as P63, cytokeratin (CK) 5/6, calponin, and smooth muscle actin (SMA) [2,3]. Papillary breast lesions comprise of intraductal papilloma (IP) with or without atypia, papillary ductal carcinoma in situ (DCIS), encapsulated papillary carcinoma (EPC), solid-papillary carcinoma (SPC), and invasive papillary carcinoma (IPC) [4].

Diagnosis is made through a variety of steps, including physical examination, ultrasonography, mammography, MRI, ductography, mammary ductoscopy, and cytological examination of the nipple discharge [5]. However, distinguishing benign from malignant papillary lesions based on imaging modalities might be challenging due to heterogeneous presentations on ultrasound, MRI, and mammography. Additionally, some benign and non-papillary lesions can mimic papillary lesions on imaging, underscoring the necessity of pathological confirmation for a definitive diagnosis [6].

We herein report a 51-year-old man complaining of hemorrhagic discharge from the left nipple, which was diagnosed as left breast atypical papillary lesion through CNB. Given the diagnostic challenges, premalignant nature, treatment considerations, and infrequency considering this case in actual clinical setting is important.

## Case presentation

A 51-year-old man presented to our breast clinic, complaining of a unilateral hemorrhagic nipple discharge for months. The patient mentioned spontaneous and intermittent discharge, almost a drop, from an orifice located medial of the left nipple. There was no positive family history of breast cancer, recent trauma, systemic symptoms (fever, weight loss, fatigue), mastalgia, previous breast disease, exposure to chest irradiation, and recent use of exogenous hormones or specific medications. On physical examination, there were no visible abnormalities, including retraction, ulceration, crusting, swelling, and erythema on both nipples and areoles. No cutaneous alterations, palpable nipple, and periareolar masses or tenderness were noticed. No evidence of Lymphadenopathy was identified since no enlarged, firm, or fixed axillary and supraclavicular lymph nodes were detected.

Subsequently, pathologic and radiologic evaluations of the breast, including ultrasonography, MRI, and mammography, were conducted.

**Fig. 1:**
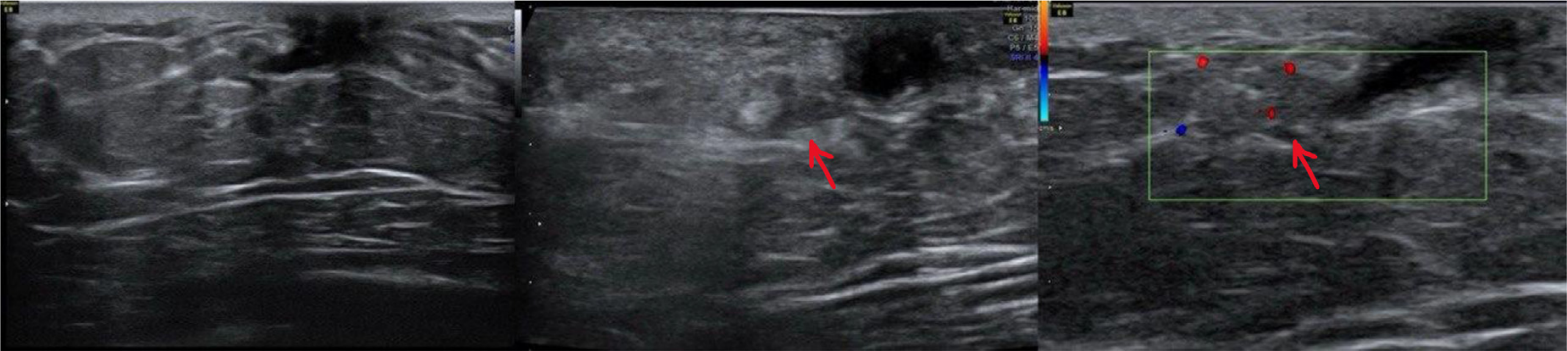
breast ultrasound shows a dilated duct in retroareolar region and an isoechoic lobulated intraductal mass (7 × 3 mm) with central vascularity in the left breast (arrow).

Ultrasonography revealed a dilated duct (up to 2.5 mm) in the retroareolar region with extension to 9-10 o’ clock region and an isoechoic lobulated intraductal mass (7×3 mm) with central vascularity was seen within the mentioned duct at a distance of about 7 mm from nipple, suggesting intraductal papilloma. There was no evidence of axillary lymphadenopathy (Fig. 1).

**Fig. 2:**
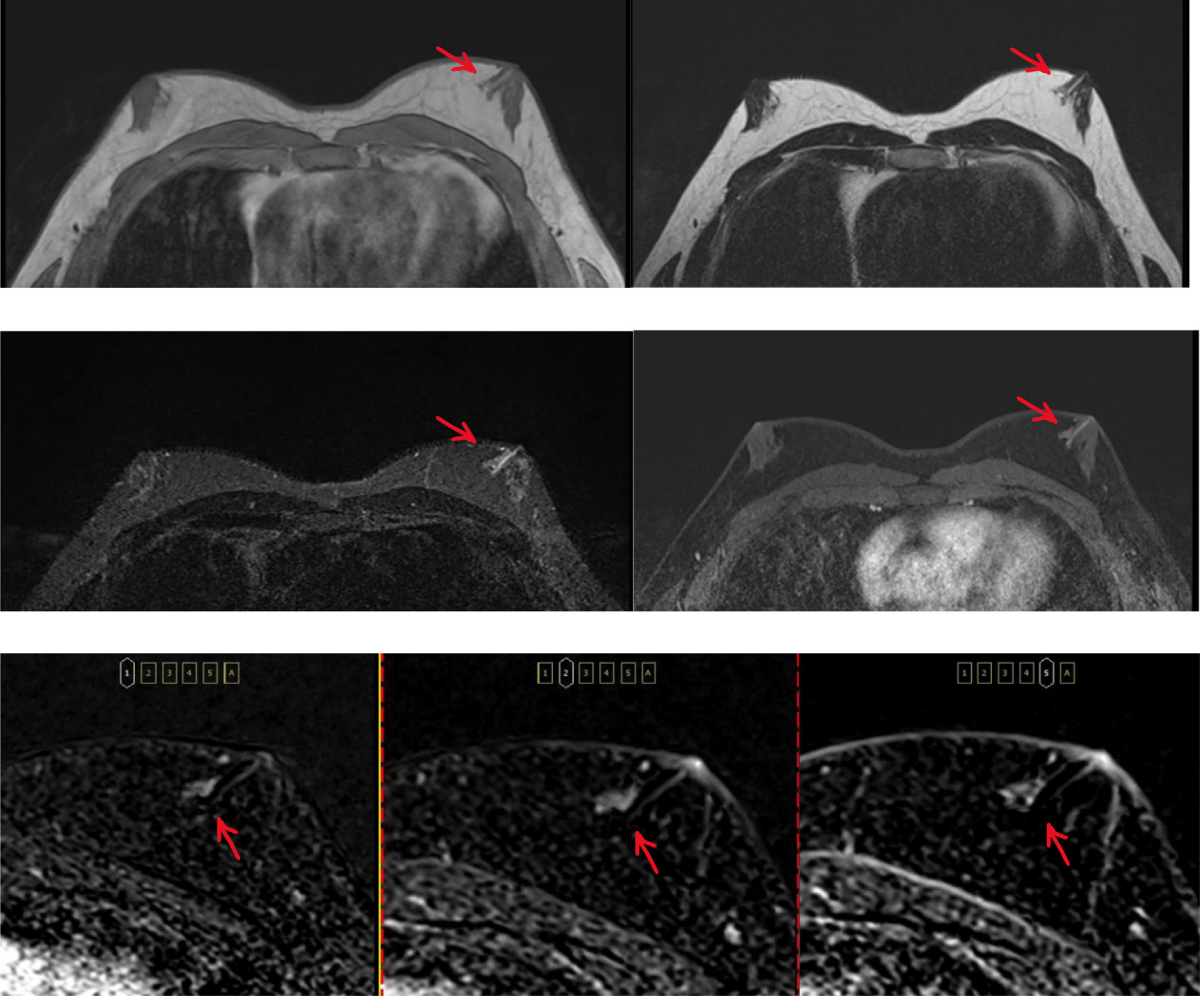
Axial MR images show a dilated duct with internal hemorrhagic products signal-intensity in the subareolar region of the left breast (arrow). First row: pre-contrast T1WI (left), T2WI (right). Second row: STIR (left), T1WI fat suppressed (right) In the left periareolar region there is an area of focal heterogeneous non-mass enhancement in the inner vicinity of the dilated duct (Third row: axial subtraction from left to right: phases 1st, 2nd, and 5th).

These findings were in conjunction with Breast MRI, which reported a dilated duct with internal hemorrhagic products signal-intensity, in the left breast, 9 o’clock subareolar region. There was an area of focal heterogeneous non-mass enhancement (11*10 mm) at the left 9 o’clock periareolar region (8 mm from nipple) and in the medial vicinity of the mentioned duct, which showed slow/progressive kinetics and mild heterogeneous T2 hyper-intensity. Evidence of bilateral mild chronic gynecomastia was seen in the subareolar parts of both breasts. There was no evidence of abnormal mass and suspicious enhancement in the anterior chest wall, and bilateral axillary lymph nodes appeared normal. The left breast was classified as BIRADS IVa (Fig. 2).

Also, bilateral full-field digital mammography showed flame-shaped glandular tissue with fat intersperse, located concentric to the nipple in subareolar regions of both breasts. All findings were in favor of symmetric gynecomastia. There is no evidence of suspicious mass, microcalcification, or any definite sign of malignancy in the breasts (BIRADS: 2, benign finding).

**Fig. 3:**
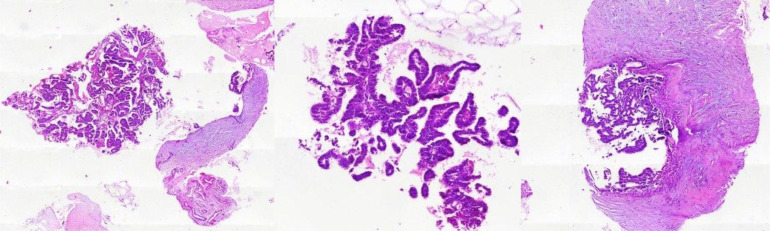
H&E staining shows Circumscribed intraductal proliferation comprised of arborizing fibrovascular cores lined by cuboidal to columnar epithelial cells with low to intermediate grade atypia.

**Fig. 4:**
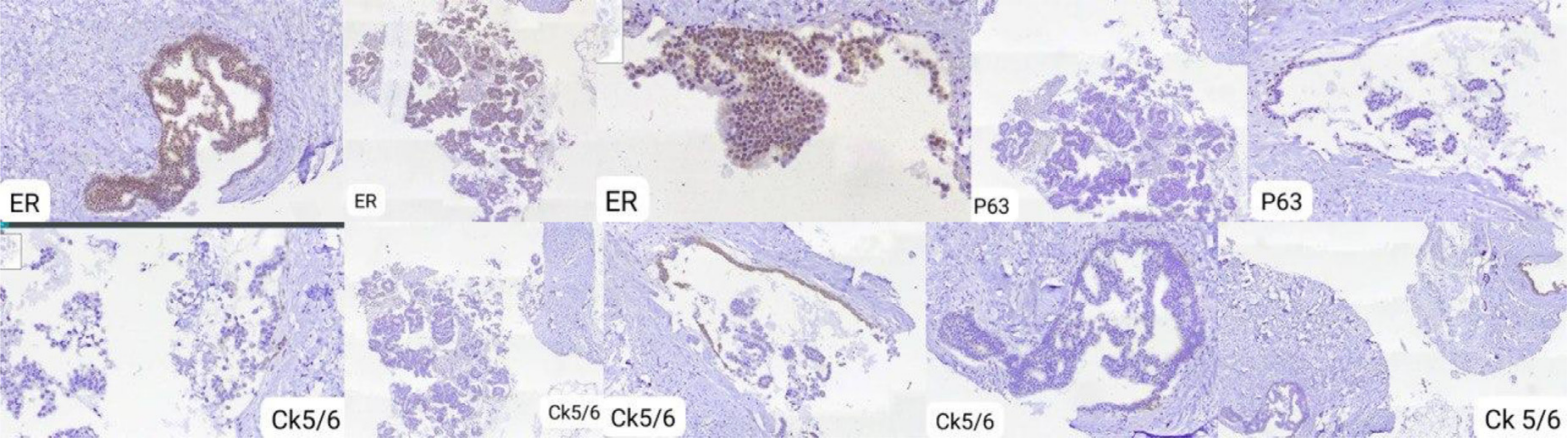
Immunohistochemistry staining shows ER (Estrogen receptor) strong and diffuse reactivity in epithelial cells, cytokeratin 5/6 (CK5/6) and P63 positive reactivity in some residual myoepithelial cells and CK5/6 negative reactivity in involved duct epithelial cells.

For the patient, a CNB was performed, and the microscopic evaluation showed a circumscribed intraductal proliferation comprised of arborizing fibrovascular cores lined by cuboidal to columnar epithelial cells with low to intermediate grade atypia (Fig. 3). IHC examinations revealed strong and diffuse reactivity of ER (Estrogen receptor) in epithelial cells, positive reactivity of cytokeratin 5/6 (CK5/6) and P63 in some residual MECs, and negative reactivity of CK5/6 in involved duct epithelial cells, supporting the diagnosis of an atypical papillary lesion such as IP with DCIS, papillary DCIS, EPC, or SPC (Fig. 4).

Following these findings, the patient underwent complete duct removal through a circumareolar incision. Surgical excision was recommended owing to the diagnosis of atypical papillary lesion on CNB, since excision is typically indicated for these lesions due to their potential correlation with malignancy and risk of underestimation during biopsy. The surgical sample was sent for pathological assessment. The diagnosis was gynecomastia with usual ductal hyperplasia, and no evidence of residual involved duct in the surgical sample, therefore IHC analysis was not repeated on the excised tissue.

The surgical specimen revealed no residual papillary lesion, only background breast tissue with gynecomastia and usual ductal hyperplasia was observed. Considering the small size of the lesion on imaging (7 × 3 mm), it is most likely that the atypical papillary lesion was entirely excised during CNB. Accordingly, the absence of residual lesion in the excised sample suggests that the atypical papillary lesion was completely removed during biopsy rather than diagnostic discrepancy.

The patient did not attend scheduled postoperative follow-up visits. Thus, long-term clinical and imaging follow-up data could not be obtained.

## Discussion

Male breast papillary lesions represent a rare subset of breast pathologies, typically affecting the major lactiferous ducts in the subareolar region, which can have hemorrhagic or sticky discharge [1,7]. Presence of papillae is considered the histological hallmark, which is composed of arborizing fibrovascular stroma. These lesions can have varying histological presentations. Some cases have equal and coordinated stromal and epithelial proliferation, whereas in some cases, the epithelial component proliferates excessively and extends into the connective tissue of the duct wall [8].

Approximately 5%-10% of cases with hemorrhagic discharge are associated with malignancy. Blood-stained secretion from a single duct, mass-associated discharge, and nipple discharge in individuals over 50 years old are considered risk factors of malignancy [9]. The assessment methods for breast atypical papillary lesions include physical examination, mammography, ductography, ultrasonography, MRI, mammary ductoscopy, and cytological examination of the nipple discharge [5]. In mammography, small papillomas are difficult to diagnose, particularly in the retroareolar region, due to the high tissue density and the minimal applied compression. In ultrasound, IPs are detected as marginated solid nodules or as nodules adherent to the duct wall inside a dilated duct. Compared to mammography and ductography, ultrasound has higher sensitivity to detect papillomas, but a negative ultrasound does not rule out an IP, especially when nipple discharge is present. When mass-related fluid is being discharged, the duct is not fully dilated, thus making the mass indetectable in sonographic evaluation [10,11]. Although useful, radiological modalities cannot distinguish benign from potentially malignant papillary lesions. In recent years, newer contrast-enhanced imaging methods have been developed to optimize mass characterization and overcome certain diagnostic limitations. Studies in female patients suggest that contrast-enhanced mammography (CEM), an emerging imaging approach that integrates mammography with intravenous contrast administration to visualize breast neovascularity, offers considerable promise for differentiating benign and malignant lesions through morphological assessment and kinetic enhancement [12,13]. Nevertheless, most evidence on CEM come from studies in female patients and research specifically focusing on its role in male breast lesions is sparse, with only a few reports documenting its use in men [14].

Moreover, the role and effectiveness of cytopathological evaluation of nipple discharge in men is still doubtful, as atypical lesions could resemble cytological features of low-grade carcinoma. Therefore, surgical resection and subsequent histopathological studies are carried out for a more accurate diagnosis [5,9,15]. Malignant papillary lesions are typically fragile structures and contain less fibrotic tissue than those seen in benign IPs. Moreover, malignant cells are associated with higher nucleocytoplasmic ratio, leading to more basophilic (bluish) appearance at low magnification. On the contrary, benign papillary lesions often tend to appear more eosinophilic (pink), which may be the consequence of apocrine metaplasia and the presence of MECs. Also, the epithelial component of malignant lesions is comprised of homogeneous cell population, whereas, benign lesions exhibit greater cellular diversity due to a mixture of epithelial, MEC, and basal cells. In benign papillary lesions, MECs are observed along the epithelial-stromal junction, both within the fibrovascular cores and at the rim of the lesion. Approximately, 80% of the EPC and SPC cases and all IPC cases lack MECs at the periphery [16].

The current study presented a case of an atypical papillary lesion of the left breast and bilateral gynecomastia in a 51-year-old man, who had experienced spontaneous and intermittent bloody discharge. Microscopic observations, also supported the diagnosis of an atypical papillary lesion. IHC markers evaluation showed CK5/6 and P63 positive reactivity in residual MECs, negative CK5/6 reactivity in involved duct epithelial cells, and strong and diffuse reactivity of ER in epithelial cells which is indicative of an atypical papillary lesion. Jorns et al. also presented a case of IP with both UDH and atypia, which demonstrated CK5/6 positive staining in UDH and loss of staining in the atypical region. They also emphasized that columnar cell hyperplasia and apocrine metaplasia, which are typically detected in IPs, may result in negative CK5/6 reactivity [17]. A key feature of our case is the absence of any residual papillary component in the surgical specimen following a CNB diagnosis of atypical papillary lesion. Given its small size, complete removal during biopsy is the most likely cause. This finding does not signify histopathologic discordance, but rather represents the known possibility of total resection of small papillary lesions in the course of biopsy.

Our IHC findings were compatible with diagnostic imaging, reporting an isoechoic lobulated intraductal mass, classified as BIRADS IVa in the left retroareolar area, as well as bilateral mild chronic gynecomastia, which could be considered as a possible cause of papillary lesions in men. In a case study, Reid-Nicholson et al. observed that three patients which were diagnosed with papillary breast lesions had a history of using gynecomastia-stimulating drugs, suggesting the possible causal relationship between gynecomastia and breast papillary lesions [18]. However, the association between gynecomastia and risk of breast cancer development remains controversial. Furthermore, hyperprolactinemia has been implicated as possibly involved in the pathogenesis of male Ips [9]. Sara et al. reported a case of a 71-year-old man admitted with IP, who had been undergoing phenothiazine (prolactin-increasing drug) treatment for over ten years due to a chronic psychiatric disorder [19].

As reported by the current UK national guidelines, breast core biopsy specimens, indicative of a papillary lesion, are categorized as “B2” (a small and accidentally- found lesion), “B3” (with unknown risk of malignancy), and “B4” (atypical or suspicious features). P J Carder et al. suggested that papillary lesions diagnosed as B2 and B3 on core biopsy may not be required to be surgically resected, emphasizing the importance of NHSBSP core biopsy reporting system. In their study, all B2 and B3 lesions were ultimately considered benign, as B3 lesions exhibited negative predictive value of 100% for malignancy, whereas all B4 lesions proved malignant and consequently demonstrated positive predictive value of 100% for malignancy. Generally, they recommended surgical excision when there is discrepancy in radiological and pathological findings, unusual or atypical histological features, and uncertainty in pathological diagnosis [20]. Their findings are in contrast to the conclusion of Kim BJY et al. which reported a case of an IP with papillary structure and considered surgical resection as a usual treatment of papilloma for both women and men, similar to our case [21]. Furthermore, Gaurav et al. reported a 52-year-old man with an initial diagnosis of IP, which was closely followed instead of surgical excision. The lesion later progressed into invasive ductal carcinoma three years later [22]. These observations indicate the need for close follow-up and in-time and accurate diagnosis for appropriate clinical decision. This case further supports the prevailing surgical approach to atypical papillary lesions. CNB may underestimate coexisting atypia or malignancy, thus, complete excision is recommended to achieve diagnostic accuracy. A limitation of this report is lack of long-term follow-up information, as the patient did not attend postoperative evaluations. Although, excised tissue did not contain residual lesion, the absence of follow-up periods, precludes a comprehensive assessment of long-term clinical outcomes.

## Conclusion

This case emphasizes on diagnostic and management challenges of atypical papillary lesions in the male breast. Complete removal of small papillary lesions through CNB may lead to the absence of residual papillary component on subsequent surgical excision. Accordingly, given the risk of potential histopathologic underestimation, surgical excision remains the standard management strategy to guarantee accurate diagnosis and patient safety.

## Patient consent

Written informed consent has been obtained from the patient in Persian (the patient's native language). Upon request, we will send it to the respected journal.

## References

[1] Zhong E., Cheng E., Goldfischer M., Hoda SA. (2020). Papillary lesions of the male breast: a study of 117 cases and brief review of the literature demonstrate a broad clinicopathologic spectrum. Am J Surg Pathol.

[2] Brogi E., Krystel-Whittemore M. (2021). Papillary neoplasms of the breast including upgrade rates and management of intraductal papilloma without atypia diagnosed at core needle biopsy. Mod Pathol.

[3] Dewar R., Fadare O., Gilmore H., Gown AM. (2011). Best practices in diagnostic immunohistochemistry: myoepithelial markers in breast pathology. Arch Pathol Lab Med.

[4] Rakha E., Sasano H., Wu Y. (2019). WHO classifcation of tumours editorial board: breast tumours. WHO Class Tumours Series.

[5] Al Sarakbi W., Worku D., Escobar P.F., Mokbel K. (2006). Breast papillomas: current management with a focus on a new diagnostic and therapeutic modality. Int Semin Surg Oncol.

[6] Eiada R., Chong J., Kulkarni S., Goldberg F., Muradali D. (2012). Papillary lesions of the breast: MRI, ultrasound, and mammographic appearances. AJR Am J Roentgenol.

[7] Singh A., Nigam J.S., Misra V., Singh PA. (2014). Diagnosis of a nonpalpable intraductal papilloma without radiological abnormality by nipple discharge smear examination: a case report. Breast Cancer (Auckl).

[8] Kulka J., Madaras L., Floris G., Lax S.F. (2022). Papillary lesions of the breast. Virchows Arch.

[9] Vagios I., Nonni A., Liakea A., Constantinidou A., Kontos M. (2019). Intraductal papilloma of the male breast: a case report and review of the literature. J Surg Case Rep.

[10] Rahman N.A., Arnaout I., Krimsti M., Mardini A., Rahme K., Ishkhanian S. (2024). Unusual presentation of intraductal papilloma on the nipple: a case report. Int J Surg Case Rep.

[11] Nguyen C., Kettler M.D., Swirsky M.E., Miller V.I., Scott C., Krause R. (2013). Male breast disease: pictorial review with radiologic-pathologic correlation. Radiographics.

[12] Jochelson M.S., Lobbes MB. (2021). Contrast-enhanced mammography: state of the art. Radiology.

[13] Xu W., Chen L., Zeng W., Xu Z., Ma M., Chen W. (2024). Addition of contrast-enhanced mammography enhancement patterns and morphology for differentiating benign from malignant papillary breast lesions. Br J Radiol.

[14] Yang M.L., Bhimani C., Roth R., Germaine P. (2023). Contrast enhanced mammography: focus on frequently encountered benign and malignant diagnoses. Cancer Imaging.

[15] Richter-Ehrenstein C., Tombokan F., Fallenberg E.M., Schneider A., Denkert C. (2011). Intraductal papillomas of the breast: diagnosis and management of 151 patients. Breast.

[16] Rakha E.A., Ellis IO. (2018). Diagnostic challenges in papillary lesions of the breast. Pathology.

[17] Jorns JM. (2016). Papillary lesions of the breast: a practical approach to diagnosis. Arch Pathol Lab Med.

[18] Reid-Nicholson M.D., Tong G., Cangiarella J.F., Moreira AL. (2006). Cytomorphologic features of papillary lesions of the male breast: a study of 11 cases. Cancer.

[19] Sara A.S., Gottfried MR. (1987). Benign papilloma of the male breast following chronic phenothiazine therapy. Am J Clin Pathol.

[20] Carder P.J., Garvican J., Haigh I., Liston JC. (2005). Needle core biopsy can reliably distinguish between benign and malignant papillary lesions of the breast. Histopathology.

[21] Kim J.Y., Jung Jung E., Kim J.M., Park T., Kim T.H., Lee J. (2024). Case report of an intraductal papilloma of the male breast with a typical bloody discharge. Ann Case Rep.

[22] Anand G., Al-Khalisi N., Arif D., Hamidpour S., Lewis T. (2018). Rare case of male breast intraductal papilloma progressing to invasive ductal carcinoma: a radiologic-pathologic correlation. Radiol Case Rep.

